# Is the treatment of ankle osteoarthritis changing over time in Italy? Analysis of temporal trends for fusion and arthroplasty in a population-based study from 2001 to 2022 on the National Hospital Discharge Record database

**DOI:** 10.1186/s10195-024-00809-8

**Published:** 2025-01-29

**Authors:** Adriano Cuccu, Elena Manuela Samaila, Enrico Ciminello, Umberto Alfieri Montrasio, Fabrizio Cortese, Stefania Ceccarelli, Tiziana Falcone, Marina Torre

**Affiliations:** 1 Italian National Registry of Implantable Prostheses (RIPI), Italian National Institute of Health, Viale Regina Elena 299, 00161 Rome, Italy; 2 Department of Statistical Sciences, “La Sapienza” University of Rome, Piazzale Aldo Moro 5, 00185 Rome, Italy; 3 Department of Orthopaedics and Trauma Surgery, University of Verona, Piazzale L. A. Scuro, 10, 37134 Verona, Italy; 4 IRCCS Galeazzi-Sant’Ambrogio Hospital, Via Cristina Belgioioso, 173, 20157 Milan, Italy; 5 Orthopaedic and Traumatology Unit, Santa Maria del Carmine Hospital, Corso Verona, 4, 38068 Rovereto, TN Italy

**Keywords:** Total ankle replacement, Ankle fusion, Registry, Epidemiology, Public health

## Abstract

**Background:**

Treatment of ankle osteoarthritis by total ankle replacement (TAR) is increasing worldwide. The aim of the study was to present the overall temporal trends of TAR throughout 22 years (2001–2022) in Italy, analyzing the distributions of hospitals by volume of activity and patients by age and sex, drawing on the National Hospital Discharge Record database. Furthermore, as a secondary aim, we compared these trends with those of ankle fusions.

**Materials and methods:**

International Classification of Diseases, 9th Revision, Clinical Modification (ICD9-CM) codes of interest were identified to browse the Italian National Hospital Discharge Record database. Surgical volumes, trends over time, classes of hospital activity volume, sex and age of patients, and population incidence rates were described. The statistical significance of time series trends was assessed by the Cox–Stuart test with randomness as a null hypothesis.

**Results:**

20,248 ankle procedures (total ankle replacements 8853 and ankle fusions 11,395) were extracted from 231,601,523 admissions registered nationally from 2001 to 2022. The yearly total number of TARs significantly increased almost tenfold from 96 to 996 (*p* < 0.05), while the number of fusions exhibited a stationary behavior (*p* > 0.05). The increased trend in TAR procedures was concentrated mostly in the North of Italy, with predominantly males between 55 and 64 years of age. The analysis of the number of procedures performed on inhabitants by region and that performed by all the hospitals in the region showed a different pattern across Italy.

**Conclusions:**

The substantial increase in TARs may be owing to improved implant designs and innovative surgical technologies, which allow the treatment of more severe cases and deformities, previously untreated or treated by a fusion. This trend highlights the need to invest in implementing high quality registries by promoting surgeons’ participation in data collection.

*Level of evidence*: population based study, level 1 evidence.

**Supplementary Information:**

The online version contains supplementary material available at 10.1186/s10195-024-00809-8.

## Introduction

End-stage osteoarthritis (OA) of the tibiotalar joint is a leading cause of pain, loss of function, disability, and decreased quality of life [1]. Post-traumatic ankle OA occurs in up to 70% of injuries [2], with other causes including rheumatoid arthritis, idiopathic arthritis, neuropathic arthritis, osteonecrosis, hemophilic arthritis, septic arthritis, and gout [3]. Traditionally, they have been treated with ankle arthrodesis after conservative management fails, but concern for adjacent joint arthritis has led to a gain in the popularity of ankle implants [4–7].

Total ankle replacement (TAR) and ankle fusion are the two primary surgical options for patients who fail conservative measures. Despite multiple comparative studies evaluating clinical outcomes, there is still a substantial debate about the superior surgical treatment for end-stage ankle arthritis. [8]. TAR was developed as an alternative to ankle fusion. While first-generation implants were fraught with unacceptably high complication rates, current-generation total ankle replacement designs are considered more biomechanically rational to mimic the motion of the native ankle joint, with better balance, mobility, and constraint and appreciation of the soft tissue ligament contribution to ankle and hindfoot function [9–16]. Additionally, these implants can be more accurately inserted, with improved instrumentation and the possibility of using patient-specific instrumentation, allowing for biological on-growth to promote long-term fixation [17]. These newer designs and refined surgical techniques have led to more favorable outcomes [12, 18, 19] with benefits including preservation of ankle motion, improved gait, and preservation of adjacent joints. Still, the long-term survivorship of fourth and fifth-generation implants remains unknown.

As the number of surgeons performing ankle arthroplasty increases, it is important to understand admissions and outcomes in the absence of quality long-term prospective trials comparing arthrodesis and arthroplasty of the ankle. Metrics, including hospitalization costs, are becoming increasingly important drivers of health care today.

The aim of this study is to present the overall temporal trends of TAR throughout 22 years (2001–2022) in Italy. To better describe the context, the distribution of hospitals by volume of activity and patients by age and sex was analyzed, drawing on the National Hospital Discharge Record (HDR) database. Furthermore, as a secondary aim, these trends were compared with those of ankle fusions.

## Materials and methods

Hospital Discharge Records (HDRs) are anonymized and made available by the Italian Ministry of Health to the Italian National Institute of Health (ISS) for epidemiological purposes. This data reports administrative, demographic, and clinical information on almost every hospitalization in the country, with a coverage reaching 99% in 2019 [20]. Procedures and diagnoses are recorded using ICD9-CM codes. Two statisticians (A.C. and E.C.) queried the HDR database, including all the records between 1 January 2001, and 31 December 2022. The following ICD9-CM codes were considered of interest for the study: 81.56 total ankle replacement and 81.11 ankle fusion. Data were retrieved in CSV format, and records of interest were queried according to the following structure: “(8156 OR 8111 ICD9-CM code is in ‘principal intervention’) OR (8156 OR 8111 ICD9-CM code is in ‘secondary interventions’).” This query strategy retrieved all records reporting at least one TAR or one ankle fusion procedure that were kept for analysis. Records reporting at least one 81.56 code among principal or secondary procedures were classified as TAR, while records reporting at least one 81.11 code were classified as ankle fusions. Procedures were counted, summing the number of records classified according to this rule.

TAR count data were also aggregated on annual basis, and temporal trends were analyzed in the whole dataset and subgroups defined by the region of hospitalization, sex, age, region of residence, and volume of activity. In total, five classes of volume of activity were defined on the basis of the number of procedures performed in the recovery institutes: 1–5, 6–20, 21–50, 51–100, and > 100. Moreover, fusion count data were aggregated annually, temporal trends were analyzed in the whole dataset, and subgroups were defined by sex.

Yearly contributions by region of hospitalization, sex, age, and volume of activity were explored and depicted. The analysis was also conducted on annual hospitalization rates × 100,000 inhabitants (HR) by region of residence, computed as [21]:

$$\text{HR}= \frac{\begin{array}{c} Number\, of\, interventions\, in\, the\, whole\, national \\ territory\, on\, patients\, resident\, in \,the\, region \end{array}}{\text{Resident population in the region}} \times \text{100,000}.$$

The volume of activity was further investigated by counting the number of hospitals falling into the volume subgroups, and temporal trend analysis was conducted.

The resident population at the national level and for each region was extracted from the Open Data portal provided by the Italian National Institute of Statistics (ISTAT) [22].

The chi-square test was performed to compare distributions by sex, and the Cox–Stuart test was carried out to assess the significance of possible monotonic trends in the selected groups. Data management and statistical analysis were performed using the software R, version 4.2.2 (2022–10–31 ucrt)—“Innocent and Trusting.”

The study was conducted following the principles of the Declaration of Helsinki. Moreover, since all the data are presented in aggregated form and the probability of identifying individuals owing to the large number of analyzed records is extremely low, no ethics committee approval was needed under national law to conduct this study.

## Results

**Fig. 1:**
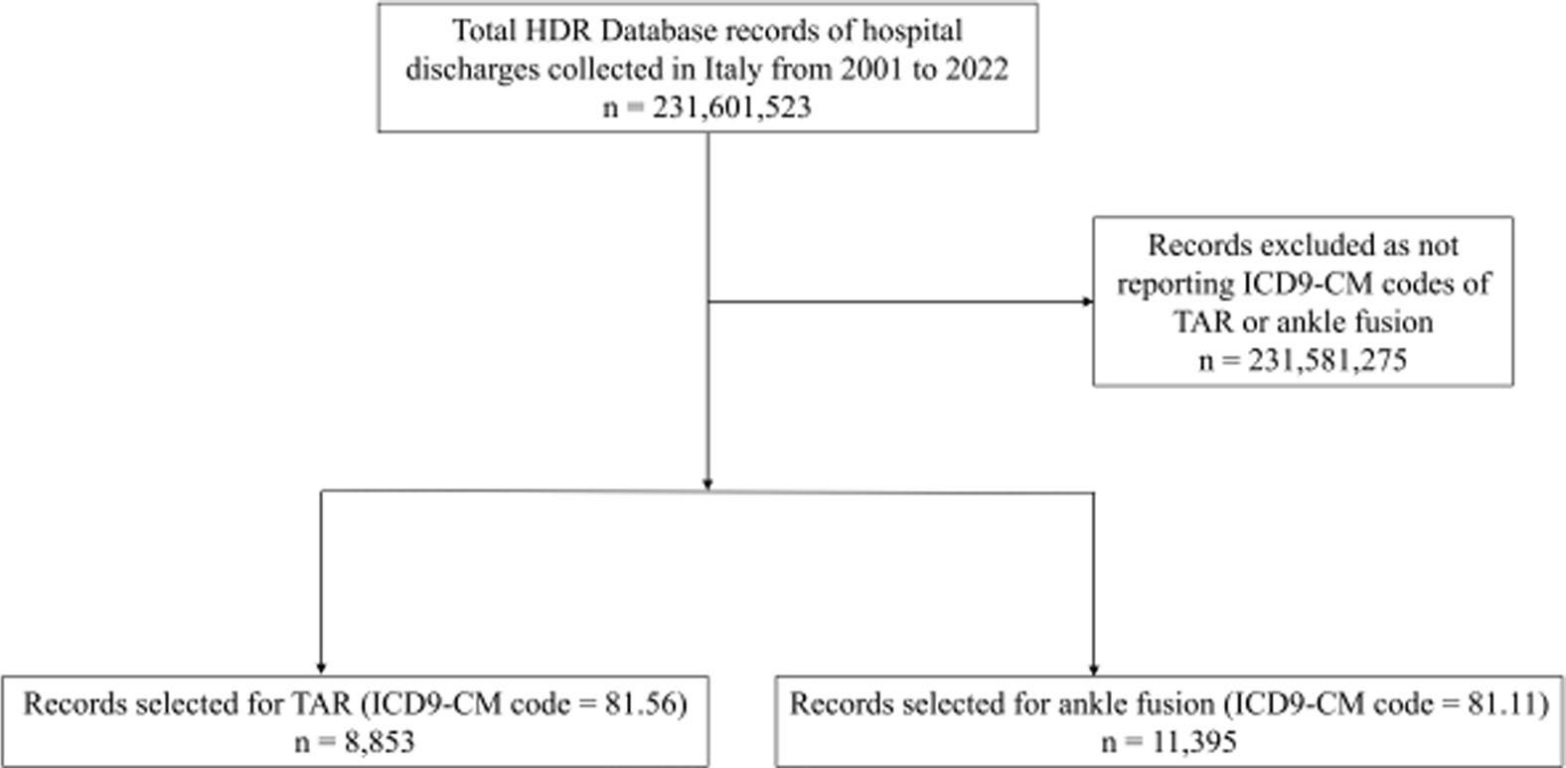
Flow chart of the data extraction process from the National HDR database

Figure 1 shows the results of the data extraction process from the 231,601,523 records included in the National HDR database. By applying the selection criteria, a total of 8853 records for TARs and 11,395 records for ankle fusions were extracted.

### Total ankle replacements

Between 2001 and 2022, 8853 TARs were performed, 46% of which were on females and 54% on males. The distributions of age classes per sex are significantly different (*p* < 0.05). The most represented age class is 55–64 years for females and < 45 years for males, and the greatest percentage difference in contribution is for the < 45 age group, which covers 16.6% of the total for females and 26% for males (Table 1).

**Table 1:** Counts of TAR procedures per age group and sex

Age classes (years)	Female		Male		Total	
	*N*	%	*N*	%	*N*	%
< 45	674	16.6	1247	26.0	1921	21.7
45–54	806	19.8	975	20.4	1781	20.1
55–64	1074	26.4	1112	23.2	2186	24.7
65–74	1039	25.6	1049	21.9	2088	23.6
75–84	447	11.0	393	8.2	840	9.5
> 84	24	0.6	13	0.3	37	0.4
Total	4064	100.0	4789	100.0	8853	100.0

Data source: Ministry of Health, National HDR database (2001–2022)

**Fig. 2:**
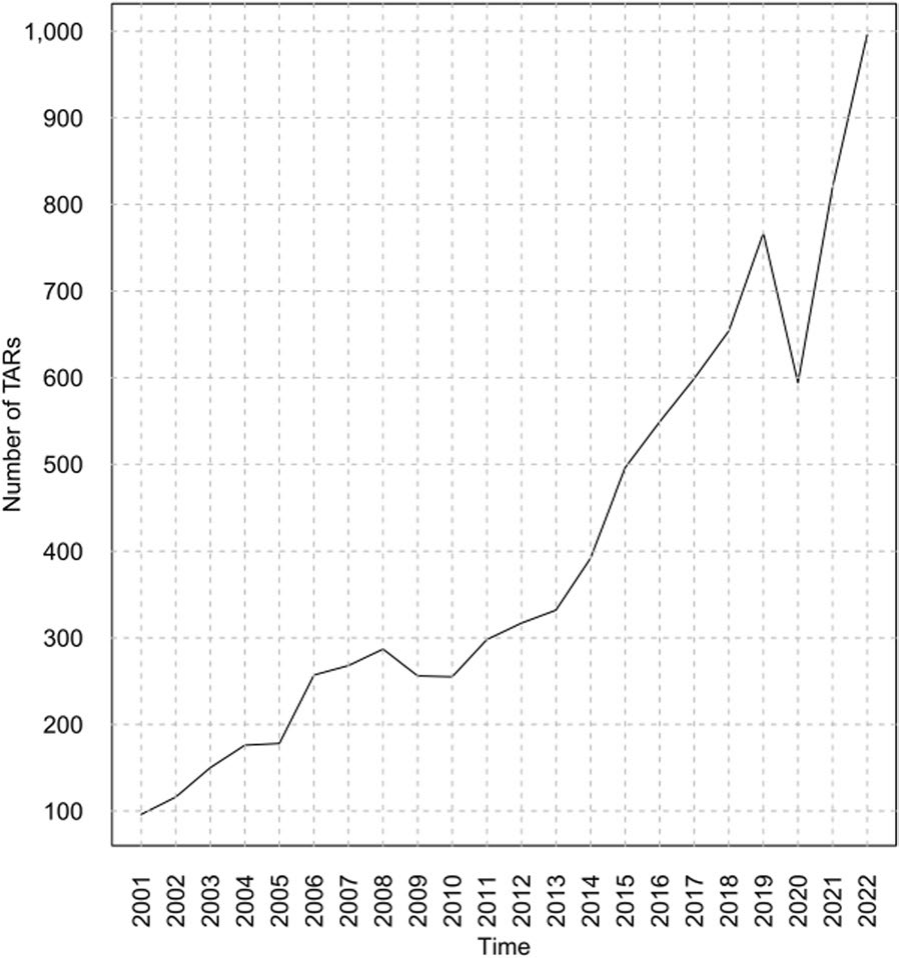
Counts of TARs performed by year. Data source: Ministry of Health, National HDR database (2001–2022)

The number of TARs performed increased from 96 in 2001 to 996 in 2022 (*p* < 0.05) with an average annual growth rate of 12.8% (Fig. 2, Supplementary material Table A1). In 2020, there was a strong decrease in the number of procedures performed, in contrast to the previous and subsequent years.

**Fig. 3:**
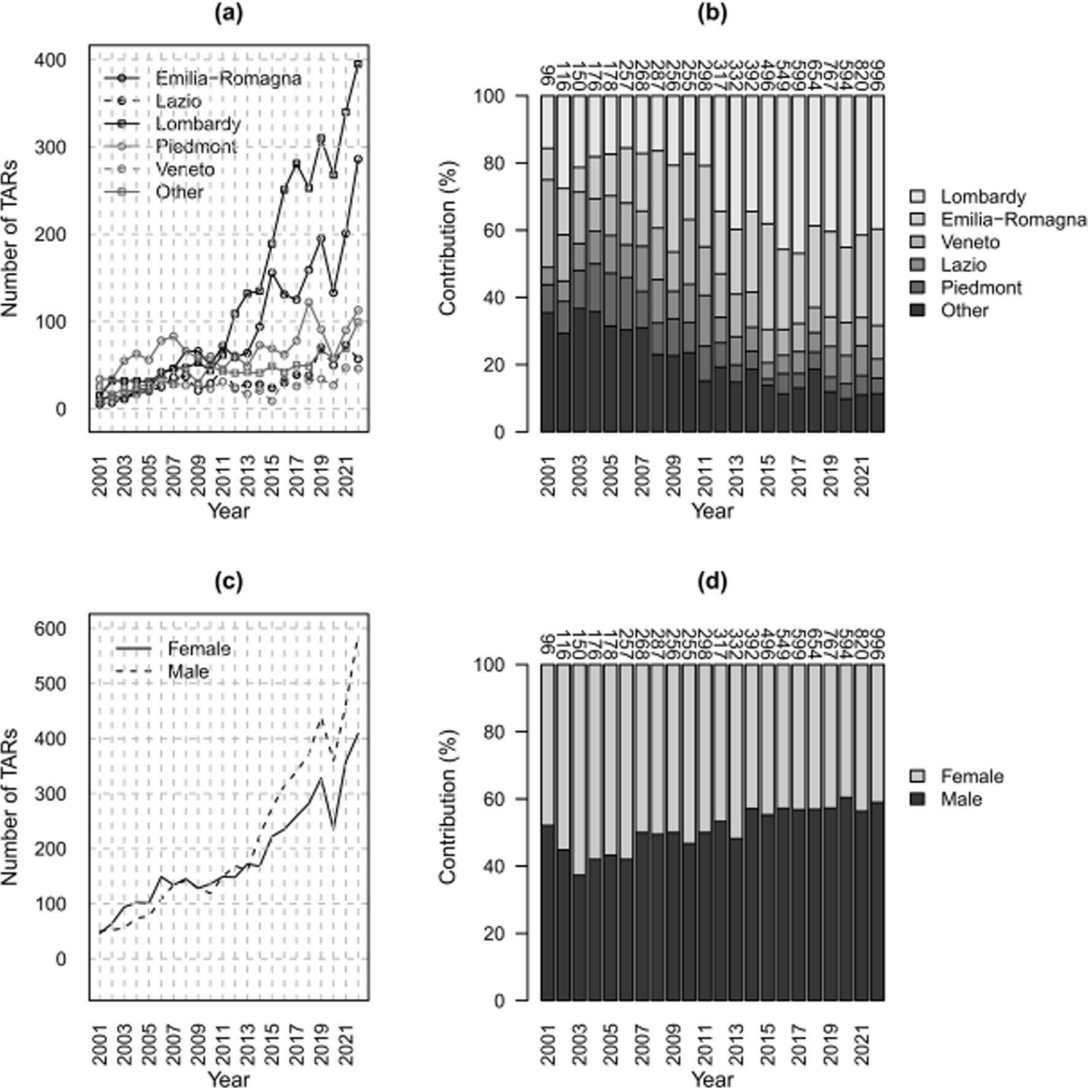
Counts of TARs (**a**, **c**) and relative contributions (**b**, **d**) per year; by region of intervention (**a**, **b**) and by sex (**c**, **d**). Data Source: Ministry of Health, National HDR database (2001–2022)

The annual number of TARs performed in Lombardy and Emilia Romagna has exceeded that of other regions since 2011 (Fig. 3a). Indeed, the contributions of all the other regions have decreased, going from around 75% in 2001 to ~ 30% in 2022, while Lombardy and Emilia Romagna have increased from around 16% and 9% in 2001 to ~ 40% and 29% in 2022, respectively, becoming the regions that have contributed the most to the overall national volume during the observed period (Fig. 3b). Going further, it turns out that the trend of the annual number of TARs was stationary in most of the regions (*p* > 0.05 for monotonic trend), increasing in seven of them (*p* < 0.05), including Lombardy and Emilia Romagna, and decreasing in Liguria (*p* < 0.05; Supplementary material Table A1). The global increase in TARs observed in the period 2001–2022 was mainly owing to that of the seven regions with an increasing trend, especially Lombardy and Emilia Romagna capturing more than half of the total number of procedures, while all the other regions but Liguria have remained nearly constant over time (*p* > 0.05 for monotonic trend). On the other hand, the annual number of TARs performed by region of residence highlights an increasing trend (*p* < 0.05) in almost all of them (Supplementary material Table A2), excluding Aosta Valley, Molise, and the Autonomous Province of Trento.

The number of TARs increased for both males and females between 2001 and 2022 (*p* < 0.05). The number of TARs for females is higher than that for males until 2014; since then, the proportion is reversed (Fig. 3c). Coherently, the proportion of intervention was higher for females until 2014, while from 2014 onward, there was a reversal of the trend whereby the contributions of males appear to be the largest, reaching approximately 59% of the total in 2022 (Fig. 3d).

**Fig. 4:**
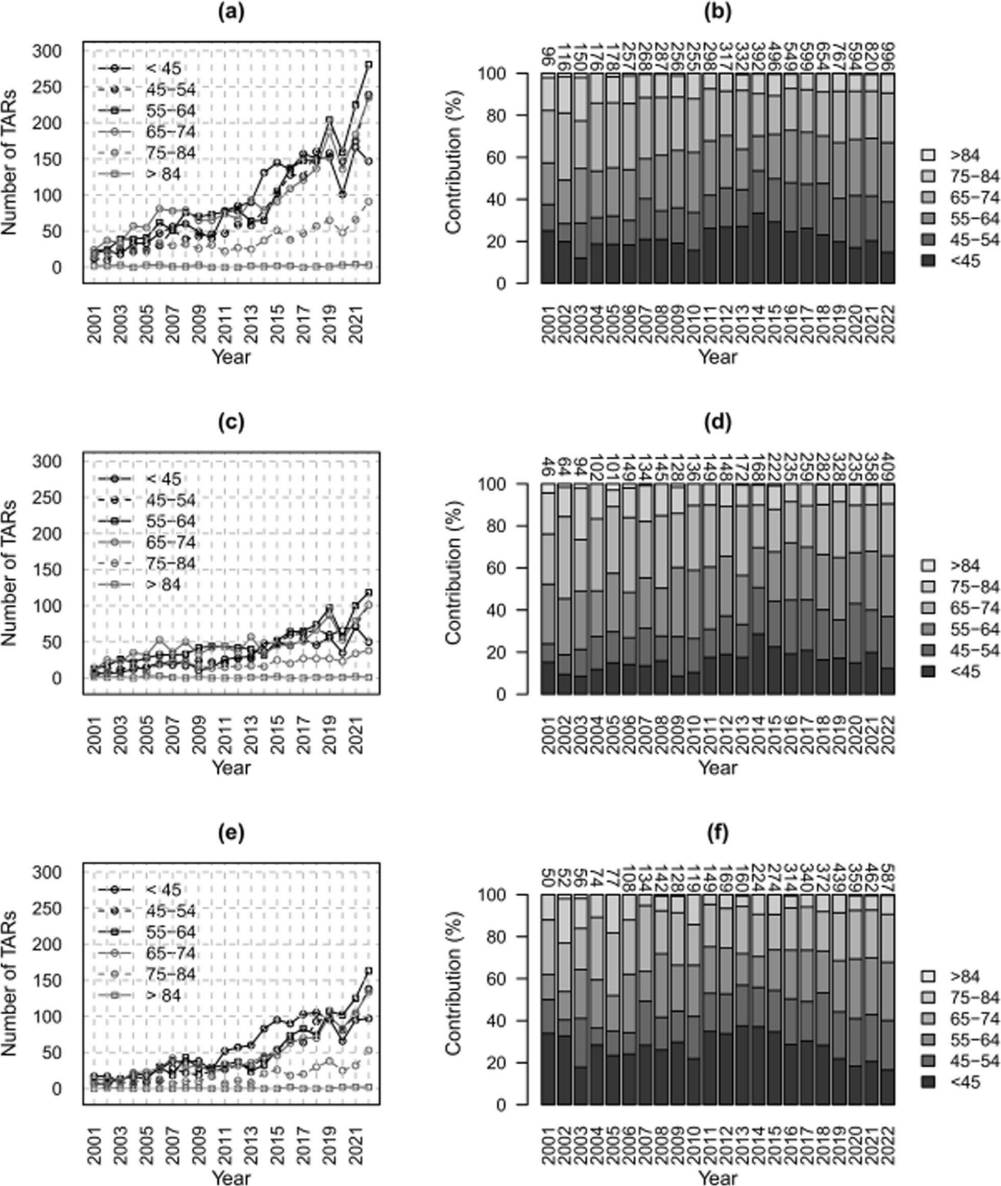
Counts of TARs (**a**, **c**, **e**) and relative contributions (**b**, **d**, **f**) per year by age classes and sex; overall (**a**, **b**), females (**c**, **d**), and males (**e**, **f**). Data Source: Ministry of Health, National HDR database (2001–2022)

The annual number of TARs increased for all age groups (*p* < 0.05), except for those older than 84 years (*p* > 0.05) (Fig. 4a, c, e). The most represented group is 55–64 years, both globally and for females, with an average annual contribution of 24% and 26%, respectively, and < 45 years for males, with an average annual contribution of 28% (Fig. 4b, d, f).

The annual hospitalization rate per 100,000 inhabitants increased significantly in Italy and in almost all the regions (*p* < 0.05), excluding Aosta Valley, Molise, and the Autonomous Province of Trento, mimicking the trend of the corresponding absolute number. Aosta Valley, the Autonomous Province of Bolzano, and Liguria had the maximum average hospitalization rate per 100,000 inhabitants (1.38, 1.31, and 1.03, respectively) (Supplementary material Table A2).

**Fig. 5:**
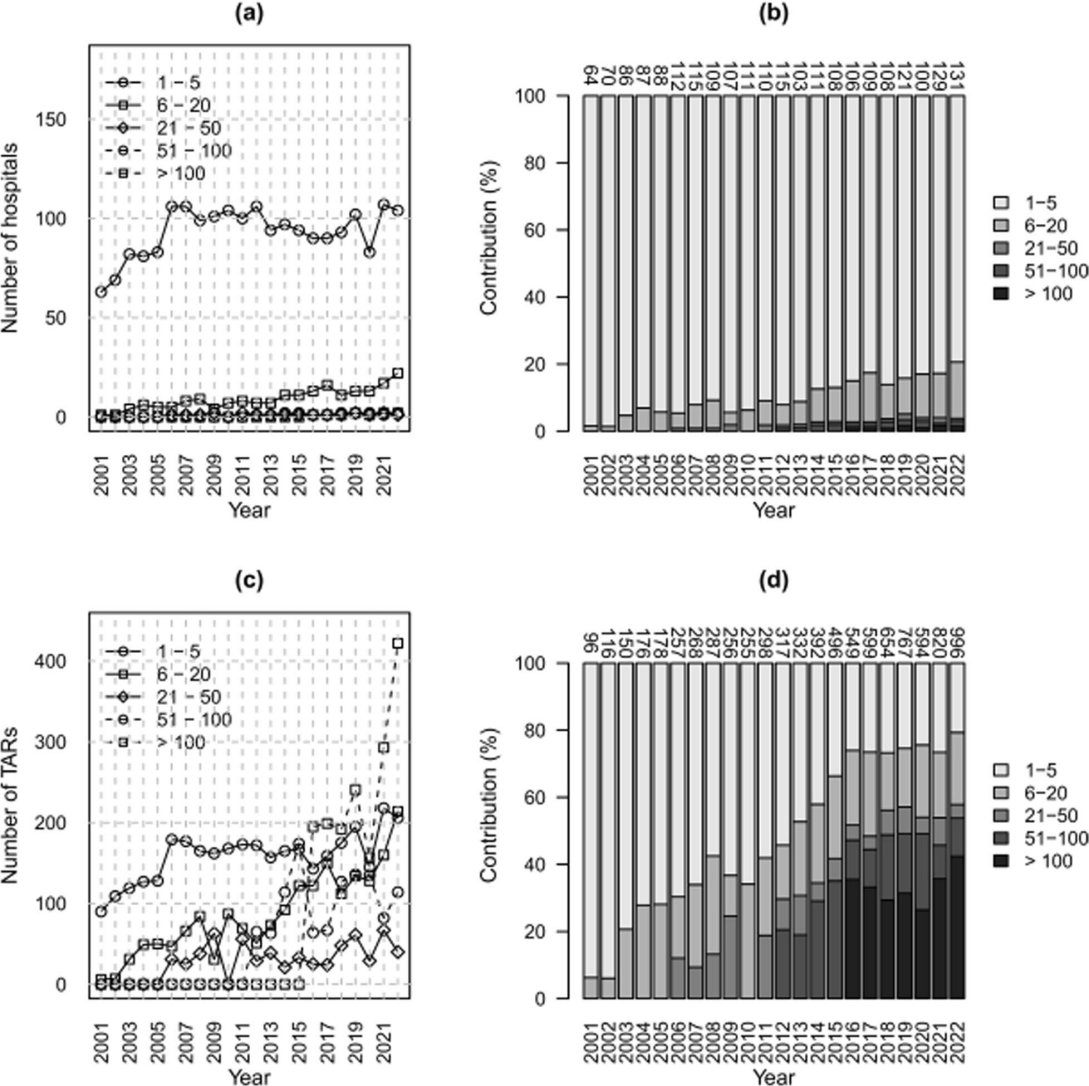
Counts of TARs (**a**, **c**) and relative contributions (**b**, **d**) per year by classes of activity volume; for hospitals (**a**, **b**) and procedures (**c**, **d**). Data Source: Ministry of Health, National HDR database (2001–2022)

The number of recovery hospitals performing TARs increased (*p* < 0.05). There was a significantly increasing behavior for those who carried out 6–20 procedures, those who carried out 51–100, and those who performed more than 100 procedures (*p* < 0.05). On the contrary, the other volume classes exhibited a stationary behavior (*p* > 0.05; Fig. 5a, b, Supplementary material Table A3). In terms of relative contributions, most of the hospitals were in classes 1 to 5. Still, the relative contribution of this class decreased from 98.4% in 2001 to 79.4% in 2022, while the relative contribution of the hospitals in classes 6–20 increased from 1.6% in 2001 to 16.8% in 2022. Starting in 2012, some hospitals performed more than 50 procedures, and in 2016, some performed more than 100 (Supplementary material Table A3).

The annual number of TARs performed by the hospitals in the volume classes 6–20, 51–100, and > 100 increased (*p* < 0.05), while for the classes 1–5 and 21–50, a stationary behavior was observed (*p* > 0.05) (Fig. 5c). Starting from 2016, most of the procedures were performed by hospitals that carried out more than 100 procedures, with a contribution ranging from around 30% to ~ 40% of the total annual number of TARs. On the contrary, the annual contribution of the hospitals performing from one to five procedures decreased from around 95% in 2001 to 20% in 2022 (Fig. 5d).

### Total ankle replacements and fusions

**Fig. 6:**
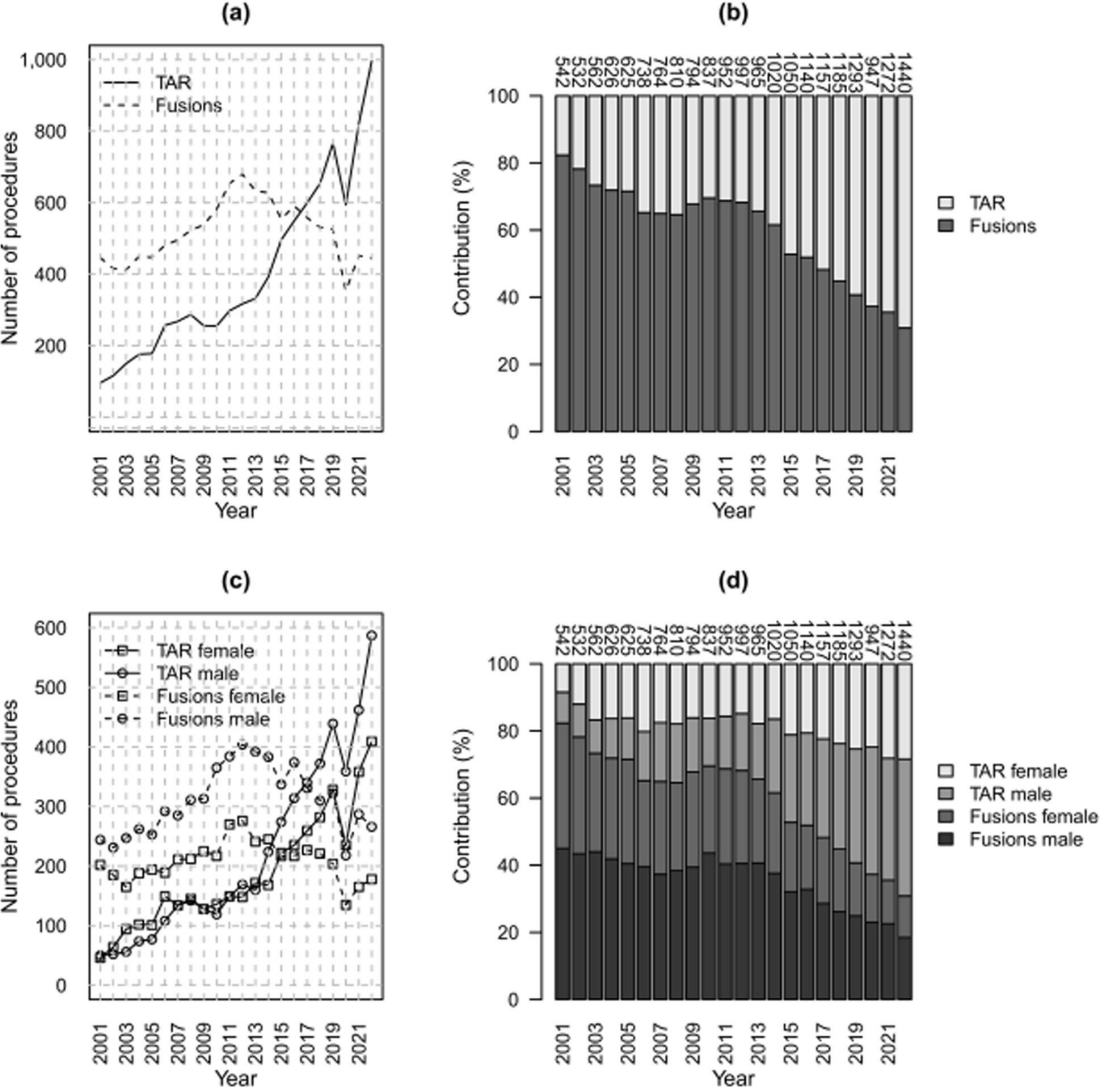
TARs and fusion counts (**a**, **c**) and relative contributions (**b**, **d**) per year; overall (**a**, **b**) and by sex (**c**, **d**). Data Source: Ministry of Health, National HDR database (2001–2022)

The comparison with fusions performed in the same period showed that, while the annual number of TARs increased between 2001 and 2022 (*p* < 0.05), the annual number of fusions was stationary (*p* > 0.05). The distribution of ankle fusions lay above that of TARs initially, while from 2017 onward, there was a reversal. Accordingly, the annual contribution of fusions decreased from ~ 80% of the total in 2001 to around 35% in 2022, while the contribution of TARs increased from 20 to 65% (Fig. 6a, b). The same situation is observed for both males and females, with an increasing distribution of TARs (*p* < 0.05) and a stationary one for fusions (*p* > 0.05) (Fig. 6c, d).

## Discussion

This study focused on investigating the overall temporal trends of TARs in Italy over the period 2001–2022. The analysis included the distribution of hospitals by volume of activity and the classification of patients by age and sex, using data from the National HDR database. A further aim was to compare these trends with those of ankle fusions. The findings highlighted that the number of TARs increased from 2001 to 2022, reaching approximately tenfold the initial number in the last year. The renewed enthusiasm for TAR likely reflects early favorable results from the newer generation prostheses.

The main result of the study was the significant increase in TARs over the 22 years analyzed. While the increase can be appreciated between 2001 and 2014, the growth clearly peaks between 2014 and 2022. This pattern, which has also been observed in other contexts, might be owing to the new generation of TAR systems marketed in 2014, which can result in up to 98% survival at 5 years and allow the treatment of OA [23, 24].

In Australia, a similar study was conducted collecting data from publicly available national registries, including the Australian Medicare Database and Australian Orthopedic Association National Joint Replacement Registry, from 2001 to 2020, finding that the incidence of ankle surgeries increased with aging and increasing comorbidities in population. Despite demonstrating no significant overall change in the ratio of TAR and fusion, there was a noticeable trend within the timeframe, with a surge favoring TAR in the last 5 years [25]. European data on TAR and AA incidences presents contrasting results. Novoa-Parra et al. reported a consistently increasing incidence of TAR in the decade between 1997 and 2007, peaking at 12.2% of combined TAR and fusions [26]. A significant decrease was registered until 2017, with TAR only representing 8.2% of all ankle surgeries. These studies hypothesized that the declining incidence of TAR in Europe was owing to concerns regarding the increasing revision rates noted with second and third-generation TAR systems. Brodeur et al. also reported an overall increase in the incidence of both TAR and AA in New York State between 2009 and 2018. Proportionally, there was a significant decrease in fusions (from 84 to 43.2%) and a corresponding increase in TAR (from 16 to 56.8%) without a decrease in total fusion volume in line with our findings [27].

In Italy, the strong decrease in procedures performed in 2020, in contrast to the increasing trend in the previous years, is owing to the reduction in elective surgical activity starting from March 2020 to fight the coronavirus disease 2019 (COVID-19) pandemic [21, 28], as well as in other countries [29, 30].

Over the 22 years under investigation, males underwent more surgeries than females. Until 2014, the number of females exceeded that of males, probably because in the first observed years, TARs were mainly recommended for patients affected by rheumatological disease (such as rheumatoid arthritis or psoriasis), which is more frequent in females [31]. From 2014 onward, the number of procedures carried out on males has always been greater than on females. Even if it has been observed that women are more susceptible to OA, there is no evidence of an association between patient sex and posttraumatic osteoarthritis (PTOA) prevalence [32]. Nevertheless, owing to the progressive marketing, in the last decades, of more advanced devices (third generation) with a better instrumentation that permitted the treatment of more posttraumatic cases [33], on the basis of our results it might be presumed that younger and sportive males affected by PTOA also had the possibility to be treated. Future studies considering also the diagnosis associated to the procedures may answer this question.

The most represented age group globally is 55–64 years. Nevertheless, the distributions differ significantly between females and males, with the most represented age classes 55–64 years and < 45 years, respectively. The proportion of the different age classes at the time of surgery varied over time; in particular, the proportion covered by the older classes decreased during the observed 20 years, confirming that younger patients required surgery to improve their quality of life and functional capacity [34]. In general, posttraumatic and osteoarthritis-related problems in the ankle affect younger patients if compared with other joints, sometimes as consequence of simple ankle sprains. Latest advancements in early diagnostics, including the use of artificial intelligence [35], and modern surgery may help to improve functional conditions and pain in the postoperative [36].

Two regions, Lombardy and Emilia-Romagna, have almost monopolized the total number of TARs performed, contributing to around 70% of the total procedures in 2022. In several cases, the increase of TARs performed on patients living in a region contrasts with the number of procedures performed in that region, which exhibited a stationary trend or even a decreasing one (Autonomous Province of Trento and, respectively, Liguria). These trends are confirmed by the analysis on hospitalization rates. This may be owing to interregional mobility, for which some people needing TAR move from their living region to a more attractive one. Indeed, Lombardy and Emilia Romagna are known to be very attractive regions also for other joint replacements [21, 37, 38], partly owing to a high volume of procedures performed in their hospitals, generally recognized as a predictor of good outcomes [39]*.* Future studies on mobility for TARs should be carried out to confirm such an assumption. About 9% of the Italian patients move outside their region to search for better treatments and accessibility to care. Inter-regional mobility is a phenomenon that affects all acute hospitalizations, with the northern and central regions attracting more than 60% of the patients escaping from the southern regions [40]. In general, data suggests that an effort by national and local administrations might be necessary to understand the causes behind such regional disparities and improve accessibility to TAR in a standardized and equitable way in the whole country, reducing territorial disparities owing to local policies, surgeons’ training, and procurement strategies.

The number of procedures performed by hospitals in each class of activity volume increased for all classes except for the one with the lowest volume. On the other hand, the overall quota of high-volume hospitals that performed TARs increased since 2014, reaching more than half of the total number of performed procedures. This may be explained by the development of some centers that have increased their volume of activity over the years and the introduction of new generation systems, which allow indication for TAR in a wider range of clinical conditions [24]. On the other hand, the majority of hospitals performing TARs still have a low volume of activity (1–5 procedures per year) but they represent only about 20% of the total number of performed replacements, a result similar to that observed in USA [41].

In the observed time window, the trend in performed fusions was stationary in Italy, as well as in USA from 1991 to 2010 [41]. This contrasts with what was observed for TARs, which increased over time. In fact, fusions were performed almost four times more often than TARs in 2001 but, after a reversal in proportion observed in 2017, they became less than half in 2022. This may be because many previously untreated conditions have become treatable by TAR over time owing to technological innovation. Another explanation can be found in a possibly stronger tendency of surgeons to indicate TARs instead of fusions. Moreover, the stationary trend of fusion may be owing to the ICD9-CM code structure, which does not allow for distinguishing whether a fusion is performed as a primary procedure or as treatment of a failed prosthesis. Therefore, the possible overestimation of this procedure should be investigated in future studies.

## Limitations

The main limitation of this study is strictly connected to the lack of granularity of the ICD9-CM codification, which has only one code for TAR in general without any specific code for TAR revision. Consequently, even if the results presented in this study are generally reliable, it is inherently impossible to know if the observed increasing trend is owing to an actual increase of the number of primary procedures or a dramatic increase of the number of revisions since it is not possible to distinguish if every considered procedure was referred to a first implant or to a revision. Moreover, a bias might be introduced by the administrative nature of ICD9-CM coding system itself. Coding errors, wrongly reported codes and missing codes are possible; this implies that the correctness in identifying procedures of interest cannot be validated. Both these issues underline the need to promote participation in the TAR registry in Italy. Indeed, the Italian Arthroplasty Registry (RIAP) has recently implemented the ankle data collection protocol [42]. The module dedicated to TAR was designed and implemented within the framework of the RIAP to collect specific data that allow distinguishing primary and revision procedures. However, nowadays, participation in RIAP is still voluntary and is on the basis of specific agreements signed between regions and the ISS. Currently, for TAR there is no agreement signed between ISS and Lombardy and Emilia-Romagna (the two regions that contribute the most to the yearly overall TAR national volume) and only few procedures have been collected in the other regions. Hopefully, this study and the landscape of the Italian context depicted here will encourage surgeons’ participation in RIAP.

Further development of this study might consider the analysis of the diagnoses associated with every procedure (if primary or posttraumatic OA or description of the implant failure) for a more granular analysis of procedure trends.

## Conclusions

Recent evidence showed better clinical results and higher satisfaction in people under 50 years undergoing TAR than ankle fusions, with a comparable rate of complications and survivorship. Younger people will have a higher reoperation rate, but in the meantime, they will prevent progressive degeneration of adjacent joints [43].

This study showed the increased trend of TAR procedures in Italy in the past two decades and volume contributions from seven regions mainly concentrated in the North of Italy, with predominantly males between 55 and 64 years of age. Longo et al. already observed a similar trend over the period 2001–2016 [44]. The analysis by region of the number of procedures performed on inhabitants and that performed by all the hospitals in the region showed a different pattern across Italy, highlighting interregional mobility that needs to be further investigated. Moreover, the lack of ICD9-CM codes able to distinguish between TAR primary and revisions underlined the need to improve the registry data collection.

Given the significant increase in the number of procedures worldwide, it is essential to promote international collaboration to harmonize registry data collection and to support updating the procedure coding for a more granular description of total ankle replacements. At the international level, the International Society of Arthroplasty Registries (ISAR) has been paving the way for establishing common agreements within the arthroplasty registry community [45]. The Orthopedic Data Evaluation Panel (ODEP), an independent panel of experts that objectively assesses the strength of the available evidence on the performance of medical implants, has already set up the criteria for evaluating ankle prostheses, considering up to 7 years of survival. As of 6 July 2024, the evaluation of 967 products from 49 suppliers was publicly available on the ODEP website [46]. They refer to the elbow, hip, knee, shoulder, spine, and wrist and not to the ankle. Given the dramatic increase in the number of TARs, especially in younger people, it is desirable that ankle replacement registries be fully implemented in the near future and that ankle prostheses also undergo the ODEP evaluation soon so that surgeons can provide patients with the safest implants.

## Supplementary Information

Supplementary Material 1

Supplementary Material 2

Supplementary Material 3

## Data Availability

The datasets used and/or analyzed during the current study are available from the corresponding author on reasonable request.

## Abbreviations

HDR: Hospital discharge record
HR: Hospitalization rate
ICD9-CM: International Classification of Diseases, 9th Revision, Clinical Modification
ISAR: International Society of Arthroplasty Registries
ISS: Istituto Superiore di Sanità (Italian National Institute of Health)
OA: Osteoarthritis
ODEP: Orthopedic Data Evaluation Panel
PTOA: Post-traumatic osteoarthritis
RIAP: Registro Italiano ArtroProtesi (Italian Arthroplasty Registry)
TAR: Total ankle replacement
